# Association of discharge policy with the length of hospital stay among patients with coronavirus disease 2019: a cross-sectional study

**DOI:** 10.3325/cmj.2022.63.71

**Published:** 2022-02

**Authors:** Kamil Polok, Karolina Górka, Tomasz Stachura, Natalia Celejewska-Wójcik, Sabina Lichołai, Katarzyna Bućko-Głód, Gabriela Adamus, Anna Kozłowska, Aleksandra Baraniec, Olga Buczma, Krzysztof Sładek

**Affiliations:** 1 Department of Pulmonology, Jagiellonian University Medical College, Kraków, Poland; 2 Centre for Intensive Care and Perioperative Medicine, Jagiellonian University Medical College, Kraków, Poland; 3 Department of Pulmonology and Allergology, University Hospital, Kraków, Poland; 4 Division of Molecular Biology and Clinical Genetics, Department of Medicine, Jagiellonian University Medical College, Kraków, Poland; Polok et al: Association of discharge policy with the length of hospital stay among patients with COVID-19

## Abstract

**Aim:**

To assess the association between discharge policy and hospital stay length, and to evaluate the factors related to duration of viral clearance among patients with coronavirus disease 2019 (COVID-19).

**Methods:**

This cross-sectional study enrolled consecutive patients aged ≥18 years with SARS-CoV-2 infection confirmed by reverse transcription polymerase chain reaction test who were admitted to hospital. The participants were divided into the test-based (TB) policy group or symptom-based (SB) group depending on the policy valid at their hospital discharge. Multivariable analyses were performed to assess the factors related to the duration of hospital stay and viral clearance.

**Results:**

The study involved 305 patients (66.6% men). The mean age was 60.9 (15.2) years. TB and SB policy groups consisted of 145 (47.5%) and 160 patients (52.5%), respectively. The TB group had significantly longer duration of hospital stay (21.0 vs 16.0, *P* = 0.003). In multivariable analysis, SB policy was associated with significantly shorter hospital stay (β-coefficient -5.87, 95% confidence interval [CI] -9.78 to -1.96, *P* = 0.003). Longer viral clearance was associated with older age (β-coefficient 0.33, 95% CI 0.15 to 0.51, *P* < 0.001) and history of cough in the pre-hospital phase of the disease (5.96, 95% CI 0.64 to 11.29, *P* = 0.028).

**Conclusion:**

SB discharge policy is preferable in the context of limited resources during the COVID-19 pandemic.

The coronavirus disease 2019 (COVID-19) pandemic has overburdened national health care systems on a scale not experienced since the Spanish flu pandemic in 1918-1920 (1-3). Between November 2019 and February 2022, over 405 million people worldwide were diagnosed with the disease and more than 5.8 million people died. An enormous effort by clinicians and researchers since the beginning of the pandemic resulted in the development of life-saving treatments and effective vaccines (1-7).

Duration of SARS-CoV-2 viral clearance affects isolation length. It may also impede patients` access to further medical care (8) since many outpatient and inpatient facilities require a negative reverse transcription polymerase chain reaction (RT-PCR) test for admission. This may considerably delay the diagnosis and management of time-sensitive and life-threatening diseases. Therefore, there is a need to identify factors associated with prolonged viral shedding.

During a crisis, health care systems need to optimally manage limited human and equipment resources (9). During the COVID-19 pandemic, various policies have been suggested regarding discharge of patients from isolation and hospital wards. In Poland, the policy based on obtaining two consecutive negative RT-PCR tests was in September 2020 replaced by a policy based on the presence and duration of symptoms. We hypothesized that discharge policy was associated with patients` outcomes, particularly the length of hospital stay. We aimed to assess the association between the two discharge policies and duration of hospital stay in patients with COVID-19. Additionally, we evaluated the factors related to the time of viral clearance.

## Patients and methods

### Study design

This cross-sectional study was performed between March and October 2020 in a single tertiary center (Department of Pulmonology and Allergology, University Hospital in Kraków). The patients signed a written consent for study participation. The study protocol complied with the Declaration of Helsinki and was approved by the Jagiellonian University Ethics Committee (KBET 1072.6120.145.2020).

### Patients and data collection

The study enrolled consecutive patients aged ≥18 years admitted to the hospital with SARS-CoV-2 infection confirmed by an RT-PCR test. The exclusion criterion was transfer from the intensive care unit (ICU). We gathered demographic and clinical information based on an interview and an analysis of medical records. We also recorded the results of laboratory tests and imaging studies performed on hospital admission. In-hospital mortality, ICU length of stay, and hospital stay length were assessed.

### Subgroups and outcomes

Patients were divided into two groups depending on the policy valid at hospital discharge. From March 2020 until September 1, 2020, Polish regulations stipulated that patients with confirmed COVID-19 can be released from isolation after receiving two consecutive negative nasopharyngeal-swab RT-PCR tests for SARS-CoV-2 at an interval of at least 24 hours (test-based [TB] policy). Patients were discharged from hospital when they met the mentioned criterion or when they were able to isolate themselves from healthy housemates. We recorded viral shedding time, defined as the interval from symptoms onset to receiving two consecutive negative RT-PCR tests within 24 hours. Viral shedding was assessed solely from nasopharyngeal swabs and not from other viral RNA sources, such as stool samples. One of the authors contacted the patients discharged with a positive RT-PCR test by phone or checked the national database to gather information about the date of release from isolation. According to the local protocol, the first RT-PCR test was performed seven days after hospital admission. If the result was positive or unclear, the patient was re-tested every seventh day until a first negative result. If the test was negative, the patient was retested on the following day.

Since September 1, 2020, patients were released from isolation or discharged after at least three days from symptoms resolution and at least 13 days from symptoms onset (symptom-based [SB] policy). Isolation of immunocompromised patients could be prolonged at the clinician's discretion. Patients were discharged if they met the mentioned criteria or were able to isolate themselves from healthy housemates. In this group, control RT-PCR tests were not routinely performed.

For some analyses, patients were divided according to disease severity into non-severely ill (patients who did not develop respiratory failure in the course of COVID-19), severely ill (patients who developed respiratory failure but were not transferred to ICU), and critically ill (patients who required transfer to ICU due to respiratory failure).

### Statistical analysis

Qualitative variables are presented as counts (%), while quantitative variables are presented as medians (interquartile range) or means (standard deviation). Continuous variables were compared by using the *t* test or Mann-Whitney U test as appropriate. Correlation between non-parametric variables was assessed by using the Spearman Rank test. A multivariable analysis of factors associated with the length of hospital stay and duration of viral shedding was performed by using a linear regression model. The variables included in both models were selected according to the available data and according to the authors` knowledge. Independent variables included in the analysis of factors associated with the length of hospital stay were discharge policy, age, development of respiratory failure throughout the hospital stay, obesity, hypertension, baseline Modified Early Warning Score (MEWS), and steroid administration. Independent variables included in the analysis of factors associated with the duration of viral shedding were age, history of cough in the pre-hospital phase of the disease, body mass index (BMI), development of respiratory failure throughout the hospital stay, diabetes, hypertension, presence of lesions in the chest x-ray, and baseline C-reactive protein (CRP) concentration. This was a complete-case analysis. A two-sided P value <0.05 was considered statistically significant. The analysis was performed with R, version 3.6.0 (R Project, Vienna, Austria).

## RESULTS

### Study population

The study enrolled 305 patients (203 or 66.6% men). The mean age was 60.9 (15.2) years. TB policy was applied in 145 patients (47.5%), while SB policy was applied in 160 patients (52.5%). Selected demographic, clinical, and laboratory characteristics are presented in Table 1.

**Table 1:** Baseline characteristics of test-based (TB) and symptoms-based (SB) discharge group*^†^

Characteristics	Entire cohort (n = 305)	TB policy (n = 145)	SB policy (n = 160)	P
Age; mean (SD)	60.9 (15.2)	60.6 (16.6)	61.1 (13.8)	0.773
Female sex	102 (33.4)	56 (38.6)	46 (28.7)	0.089
BMI, kg/m^2^	28.1 (25.2 to 31.8)	27.8 (25.1-31.1)	28.7 (25.7-32.6)	0.094
MEWS	2.00 (1.0-2.0)	1.0 (0.25-2.0)	2.0 (1.0-3.0)	<0.001
Interval between symptoms onset and hospital admission (days)	7.0 (4.0-9.0)	6.0 (4.0-9.0)	7.0 (4.0-9.0)	0.324
Respiratory failure on admission	175 (57.4)	53 (36.6)	122 (76.2)	<0.001
Hypertension	168 (55.1)	75 (51.7)	93 (58.1)	0.314
Atrial fibrillation	26 (8.5)	16 (11.0)	10 (6.2)	0.197
Congestive heart failure	41 (13.4)	21 (14.5)	20 (12.5)	0.735
Coronary artery disease	50 (16.4)	22 (15.2)	28 (17.5)	0.694
Dyslipidemia	75 (24.6)	31 (21.4)	44 (27.5)	0.269
Diabetes	81 (26.6)	33 (22.8)	48 (30.0)	0.194
Asthma	30 (9.8)	15 (10.3)	15 (9.4)	0.927
COPD	16 (5.3)	10 (6.9)	6 (3.8)	0.337
History of smoking	91 (31.6)	39 (28.5)	52 (34.4)	0.336
WBC count (10^3^/μL)	6.8 (5.1-9.8)	5.9 (4.5-7.8)	8.4 (6.0-10.7)	<0.001
D-dimer (mg/L)	0.95 (0.52-1.86)	0.90 (0.47-1.60)	0.98 (0.56-2.36)	0.027
LDH (IU/L)	363.0 (261.8-505.5)	292.0 (235.5-379.5)	430.0 (325.0-565.0)	<0.001
CRP (mg/L)	73.4 (27.1-148.5)	43.1 (11.0-94.0)	97.2 (53.1-174.5)	<0.001
Procalcitonin (ng/mL)	0.07 (0.02-0.20)	0.05 (0.02-0.13)	0.09 (0.03-0.26)	0.002
Interleukin-6 (pg/mL)	32.91 (11.63-89.87)	17.8 (1.5-54.1)	47.34 (20.5-101.4)	<0.001
NT-proBNP (pg/mL)	291.0 (121.5-1197.3)	244.0 (86.5-994.5)	384.0 (150.5-1462.8)	0.005

*Abbreviations: BMI – body mass index; COPD – chronic obstructive pulmonary disease; CRP – C-reactive protein; LDH – lactate dehydrogenase; MEWS – modified early warning score; NT-proBNP – N-terminal pro-B-type natriuretic peptide; WBC – white blood cell.

†Qualitative data are presented as n (%), while qualitative data are presented as median (IQR) unless otherwise specified.

### Comparison of discharge policies

Patients in the SB group more often presented with respiratory failure, had higher MEWS on admission, higher baseline indicators of inflammation (white blood cell [WBC] count, CRP, and procalcitonin), and higher lactate dehydrogenase level, D-dimer, and N-terminal pro-brain natriuretic peptide (NT-proBNP). The groups did not significantly differ in sex, age, BMI, and comorbidities (Table 1).

**Figure 1:**
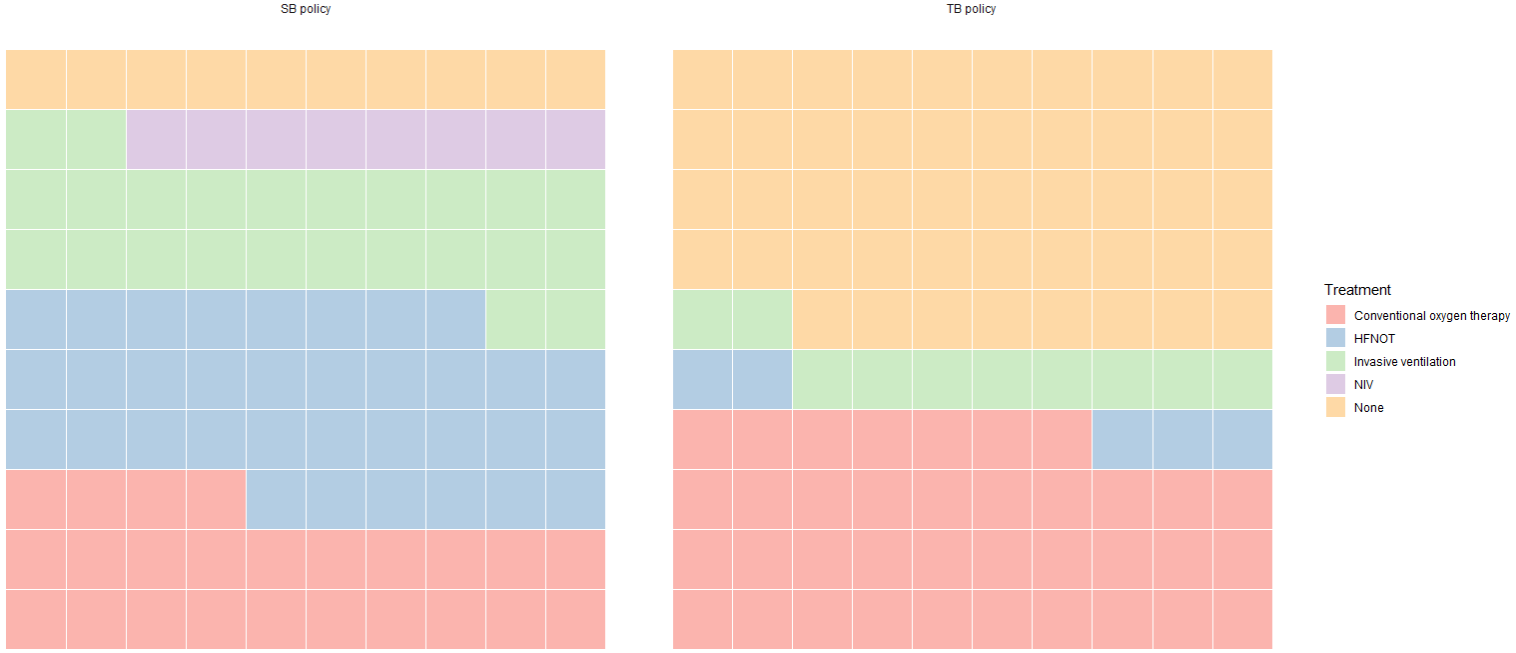
Respiratory support need stratified by the study group. HFNOT – high-flow nasal oxygen therapy; NIV – non-invasive ventilation; SB – symptom-based; TB – test-based.

**Figure 2:**
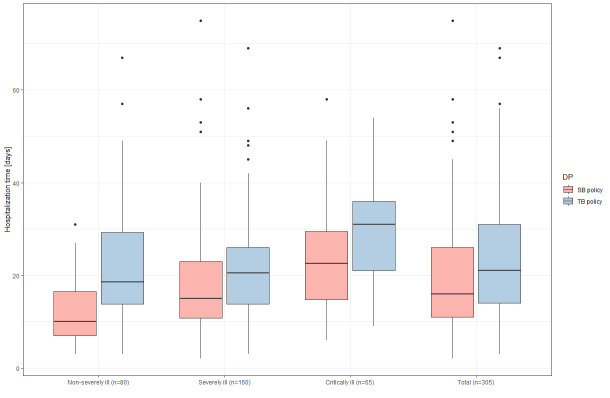
Hospital stay length according to the disease severity. DP – discharge policy; SB – symptom-based; TB – test-based.

Systemic steroids, remdesivir, and convalescent plasma were more commonly administered in the SB policy group, while chloroquine was more often administered in the TB group (Table 2). Patients in the SB group more often required advanced respiratory support (Figure 1). The groups had a similar mortality rate and ICU length of stay, but the TB group had significantly longer duration of hospital stay (21.0 vs 16.0, *P* = 0.003). A similar relationship was observed in patients with non-severe (18.5 vs 10.0, *P* = 0.007), severe (20.5 vs 15.0, *P* = 0.0498), and critical disease (31.0 vs 22.5, *P* = 0.041) (Figure 2).

**Table 2:** Treatment, respiratory support modalities, and main clinical outcomes in test-based (TB) and symptoms-based (SB) discharge group*^†^

Characteristics	Entire cohort (n = 305)	TB policy (n = 145)	SB policy (n = 160)	P
**Disease severity**				
non-severe	80 (26.2)	64 (44.1)	16 (10.0)	<0.001
severe	160 (52.5)	56 (38.6)	104 (65.0)	
critical	65 (21.3)	25 (17.2)	40 (25.0)	
Chloroquine	37 (12.1)	37 (25.5)	0 (0.0)	<0.001
Systemic steroids	158 (51.8)	25 (17.2)	133 (83.1)	<0.001
Remdesivir	83 (27.2)	0 (0.0)	83 (51.9)	<0.001
Convalescent plasma	15 (4.9)	1 (0.7)	14 (8.8)	0.003
Conventional oxygen therapy	218 (71.5)	75 (51.7)	143 (89.4)	<0.001
High-flow nasal oxygen therapy	119 (39.0)	19 (13.1)	100 (62.5)	<0.001
Non-invasive mechanical ventilation	18 (5.9)	0 (0.0)	18 (11.2)	<0.001
Invasive mechanical ventilation	52 (17.0)	14 (9.7)	38 (23.8)	0.002
Death	57 (18.7)	21 (14.5)	36 (22.5)	0.1
Duration of viral shedding (days)*	34.0 (25.0-42.0)	34.0 (25.0-42.0)	29.5 (26.5-38.0)	0.757
Length of hospitalization (days)	18.0 (12.0-28.0)	21.0 (14.0-31.0)	16.0 (11.0-26.0)	0.003
Length of intensive care unit stay (days)	10.0 (7.0-16.0)	10.0 (4.0-16.0)	10.5 (7.0-16.3)	0.348

*Data were available for 125 patients (121 patients in the TB group and 4 patients in the SB group).

**†**Qualitative data are presented as n (%) and qualitative data as median (interquartile range).

In multivariable analysis, SB policy was associated with significantly shorter hospital stay (β-coefficient -5.87, 95% CI -9.78 to -1.96, *P* = 0.003). Older age was associated with longer hospital stay (β-coefficient 0.13, 95% CI 0.03 to 0.24, *P* = 0.012). Duration of hospital stay was not associated with respiratory failure, obesity, hypertension, baseline MEWS, and steroid administration.

### Factors associated with time to viral clearance

**Figure 3:**
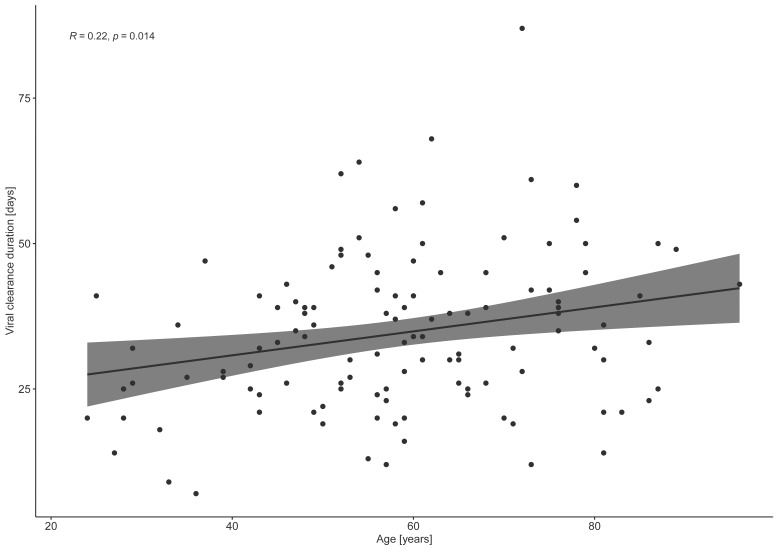
The correlation between age and duration of SARS-CoV-2 shedding (Spearman R).

Among 125 patients with available data on the duration of viral shedding, the median time from symptoms onset to achieving two consecutive negative RT-PCR tests was 34.0 days (IQR 25.0 to 42.0; ranged 7.0-87.0 days). Of these patients, 61 patients (48.8%) were non-severely ill, 44 patients (35.2%) were severely ill, and 20 patients (16.0%) were critically ill. Age significantly yet weakly correlated with viral shedding duration (Figure 3). Among survivors from the TB group, the proportion of patients discharged with a positive RT-PCR was 46.8% (58/124) in the entire study group, 59.4% (38/64) among patients with non-severe disease, 36.4% (16/44) among patients with severe disease, and 25% (4/16) among patients with critical disease. A multivariable analysis revealed that longer viral clearance was associated with older age (β-coefficient 0.33, 95% CI 0.15 to 0.51, *P* < 0.001) and history of cough in the pre-hospital phase of the disease (5.96, 95% CI 0.64 to 11.29, *P* = 0.028). Viral clearance duration was not significantly associated with BMI, acute respiratory failure, diabetes, hypertension, presence of lesions in the chest x-ray, and baseline CRP concentration.

## DISCUSSION

In this cross-sectional study enrolling more than 300 patients hospitalized because of COVID-19, TB discharge policy was associated with longer hospital stay compared with SB policy regardless of disease severity. Age and history of cough in the pre-hospital phase of the disease were independently associated with the duration of viral clearance.

The approach to isolation discontinuation has evolved with the increase in knowledge about the infectivity of COVID-19 patients. Initially, major health care organizations, including the Centers for Disease Control and Prevention and the World Health Organization, recommended the TB approach (10,11). Detection of SARS-CoV-2 RNA does not necessarily imply the presence of an infectious and active virus, and the infectivity is highest within five to six days from symptoms onset (12). Furthermore, in immunocompetent patients the risk of disease transmission after nine days from symptoms onset was very low (13-15). Accumulating evidence led to a recommendation that isolation is discontinued based on SB criteria, which were also introduced in Poland (16). Intuitively, the latter approach has the advantage of improving the patient flow in overloaded wards, and of reducing the number of tests and medical care costs. On the other hand, in some patient groups, particularly among immunocompromised individuals, the replication-competent virus can be detected even beyond 20 days after the symptoms onset (17). Viral clearance should be confirmed, eg, in patients discharged to long-term care or rehabilitation facilities or in those who cohabitate with immunocompromised individuals. Unfortunately, only a few reports have assessed the relation between discharge policy and patient-centered outcomes in the context of patients` characteristics and COVID-19 severity.

Patients in the SB group spent a median of six days fewer in the hospital than those in the TB group. However, the analysis of hospital stay length is susceptible to confounding variables including demographic characteristics, comorbidities, disease severity, and treatment. In the current study, the groups were balanced in terms of sex, age, and major comorbidities. However, patients in the SB group were more severely ill, which was indicated by a higher incidence of respiratory failure and increased need for advanced respiratory support. One would expect such patients to require more time to recuperate and to be discharged from hospital (2,18). On the other hand, administration of steroids and remdesivir and greater experience of the medical personnel and greater vigilance in venous thromboembolism prophylaxis and diagnosis had a potentially opposite effect on hospital stay length in the SB policy group (5,19,20).

To account for the mentioned imbalances, we performed a multivariable analysis to identify factors associated with an increased length of hospital stay. After adjustment for potential confounders, including demographic characteristics, selected comorbidities, MEWS on admission, presence of respiratory failure, and steroid administration, we confirmed that SB discharge policy was independently associated with a significantly shorter hospital stay, while older age was associated with a significantly longer hospital stay. Previous studies suggested that factors associated with the length of hospital stay were age >60 years, diabetes, fever, neutrophil and lymphocyte count, hypocalcemia, hypochloremia, increased CRP and D-dimer concentrations, bilateral pneumonia, and steroids administration. However, these studies were limited by small study samples and retrospective design (21-24). In our opinion, the presented results confirm the superiority of SB discharge policy over TB discharge policy, which is reflected in a shorter hospital stay without significant difference in in-hospital mortality.

After the initial hospital admission, some convalescents require further diagnostic or therapeutic procedures. Many non-COVID wards performing these procedures require a negative RT-PCR test from electively admitted patients. In this context, we identified factors associated with the duration of viral clearance. Multivariable analysis revealed that older age and history of cough before admission were associated with longer viral clearance. Surprisingly, radiographic changes on the chest x-ray and acute respiratory failure in the course of COVID-19 were not associated with longer viral clearance. These results suggest that elderly patients are at increased risk of prolonged viral shedding. Age was also associated with a longer viral shedding in a meta-analysis assessing SARS-CoV-2 viral dynamics that included 79 studies with 5340 participants (16). Unfortunately, most studies included in this meta-analysis were case series, with only two cross-sectional studies, one prospective study, and one randomized controlled trial (25-28). Interestingly, in 11 analyzed studies no live virus was isolated from respiratory samples beyond day 9 (16). These findings should be taken into account by policymakers who regulate post-COVID admissions of convalescents. A law properly regulating this aspect could improve the quality of care for COVID-19 convalescents.

This study has several limitations. Due to a relatively small study sample, the number of variables included in both models was limited, which increased the risk of omitting an important confounder. Second, due to changes in discharge policy, the information about viral clearance was restricted to patients hospitalized when TB policy was in effect. Third, according to the local policy, following a positive control RT-PCR, testing was repeated after seven days, which probably led to an overestimation of the duration of viral shedding and hospital stay. Our results reflect actual strategies applied in the early phases of the pandemic and provide real-life information about their effect on hospital stay duration and the capacity of health care system. Fourth, a lack of follow-up clinical data hinders our ability to analyze the association between discharge policy and patients` outcomes in a longer term. Finally, our single-center experience limits the generalizability of the presented results.

In conclusion, the results of this study suggest that a discharge policy based on symptoms rather than on tests is associated with shorter hospital stay and should be preferred during the current pandemic. Moreover, a significant association between prolonged viral clearance and older age in patients with COVID-19 should be taken into account by policymakers regulating health care provision for convalescents.
